# Integrating Information in Biological Ontologies and Molecular Networks to Infer Novel Terms

**DOI:** 10.1038/srep39237

**Published:** 2016-12-15

**Authors:** Le Li, Kevin Y. Yip

**Affiliations:** 1 Department of Computer Science and Engineering, The Chinese University of Hong Kong, Shatin, Neew Territories, Hong Kong; 2 Hong Kong Bioinformatics Centre, The Chinese University of Hong Kong, Shatin, Neew Territories, Hong Kong; 3 CUHK-BGI Innovation Institute of Trans-omics, The Chinese University of Hong Kong, Shatin, Neew Territories, Hong Kong; 4 Hong Kong Institute of Diabetes and Obesity, The Chinese University of Hong Kong, Shatin, New Territories, Hong Kong

## Abstract

Currently most terms and term-term relationships in Gene Ontology (GO) are defined manually, which creates cost, consistency and completeness issues. Recent studies have demonstrated the feasibility of inferring GO automatically from biological networks, which represents an important complementary approach to GO construction. These methods (NeXO and CliXO) are unsupervised, which means 1) they cannot use the information contained in existing GO, 2) the way they integrate biological networks may not optimize the accuracy, and 3) they are not customized to infer the three different sub-ontologies of GO. Here we present a semi-supervised method called Unicorn that extends these previous methods to tackle the three problems. Unicorn uses a sub-tree of an existing GO sub-ontology as training part to learn parameters in integrating multiple networks. Cross-validation results show that Unicorn reliably inferred the left-out parts of each specific GO sub-ontology. In addition, by training Unicorn with an old version of GO together with biological networks, it successfully re-discovered some terms and term-term relationships present only in a new version of GO. Unicorn also successfully inferred some novel terms that were not contained in GO but have biological meanings well-supported by the literature.Availability: Source code of Unicorn is available at http://yiplab.cse.cuhk.edu.hk/unicorn/.

Gene Ontology (GO)^1^ is the most widely-used biological ontology. It systematically summarizes current knowledge of gene products and their relationships across a wide range of species. GO contains standardized terms in three sub-categories, namely biological processes (BP), cellular components (CC), and molecular functions (MF). These terms are organized hierarchically in directed acyclic graphs (DAGs), which are tree-like structures that allow a node to have multiple parents, corresponding to the specialization of a term from multiple general terms. A gene can be annotated by multiple GO terms. If a gene is annotated by a GO term, it is also annotated by all its ancestral terms automatically. GO has been extensively used in various applications, such as assessing functional similarity of genes^2^,^3^,^4^, predicting gene functions^5^,^6^,^7^, and interpreting biological data^8^,^9^,^10^.

Most of the term-term relationships in GO are defined manually, assisted by text-mining of the literature. There are several limitations to this manual curation process. First, with the rapid expansion of biological knowledge, both the number and complexity of biological publications have become difficult to handle even with the help of text-mining. Second, the same biological concept can be described in different ways in different publications, which creates a challenge for different curators to represent the concept in a consistent manner. Finally, there is considerably more research on a subset of well-studied genes and their relationships, leading to unbalanced levels of detail in different parts of GO.

One complementary approach to GO construction is to infer terms and term-term relationships automatically from biological networks. This approach is attractive given the large amount and variety of network data already available, and the relative low cost of creating new networks and expanding existing ones using high-throughput experimental methods. The feasibility of inferring GO automatically from biological networks has been recently demonstrated^11^. In this study, a method called Network-eXtracted Ontology (NeXO) was proposed to cluster genes hierarchically based on their connections in the networks and subsequently transform the resulting clustering tree into a DAG. By using four types of molecular networks as input, NeXO was able to recover around 40% of the terms in GO based on an alignment of the terms in the NeXO and GO DAGs. Later, another method called Clique eXtracted Ontology (CliXO) was proposed to further improve the accuracy of the automatically constructed ontology^12^. This method identifies cliques of different sizes in an integrated biological network by progressively loosening the stringency for an edge to be drawn between two genes in the networks. Each identified clique forms a term that annotates the composing genes, and a new term becomes a parent of an existing term if the clique corresponding to the new term is a superset of the existing term. A major novelty of CliXO was its ability to use quantitative measures in the biological networks, such as the confidence score of the existence of an edge, in the ontology inference process. The best DAG constructed by CliXO achieved about 40% in both precision and recall when compared to the actual GO DAG.

These two studies clearly show that existing biological networks, though incomplete and noisy, contain useful information that can be used to automatically infer GO with a reasonable accuracy. On the other hand, one limitation of both NeXO and CliXO is that they infer DAGs purely based on the input network (either a single biological network or a network integrated from multiple biological networks), which implies that 1) they are unsupervised methods that cannot make use of the information contained in the existing GO, 2) the way of integrating the biological networks is not guaranteed to optimize the accuracy of ontology construction, and 3) given a fixed set of input networks, both methods cannot infer different DAGs specifically for the three different sub-ontologies of GO.

Here we extend these previous works by describing a semi-supervised method called Unicorn (Unification of Discordant Networks), which integrates multiple biological networks in a way tailored for inferring a particular sub-ontology of GO. The key idea is that each existing GO sub-ontology contains parts that are highly accurate and complete, which can be used as a training set to find out the best way to integrate biological networks for inferring the whole sub-ontology. The resulting DAG inferred by Unicorn is then expected to supplement parts of the sub-ontology not as well constructed. By using training data from a particular sub-ontology, the way to integrate the biological networks is specific to this sub-ontology. Unicorn is semi-supervised because it considers both the training part of GO and the natural distribution of edge weights in the biological networks during data processing and integration.

One major challenge of integrating different biological networks is their different distributions of edge weights and semantics, such as expression correlations in a co-expression network and similarity scores in a functional network. Unicorn uses a novel discretization procedure to turn edge weights into nominal values such that they are highly correlated with the gene-gene similarity values based on the training set of the GO sub-ontology. The resulting discretized values in the different networks can then be integrated easily.

We tested Unicorn by 1) evaluating its accuracy on left-out parts of the GO sub-ontologies not involved in training, 2) constructing a DAG by using an old version of GO for training, and comparing the newly discovered terms with a new version of GO, and 3) surveying the literature for supports of novel terms discovered by Unicorn that are not in existing GO. These tests showed that Unicorn can construct specific GO sub-ontologies accurately and identify biologically meaningful new terms.

One recent study has also engaged multiple biological networks to infer gene ontology in a supervised manner^13^. Our work is fundamentally different from it in that the method in this study does not attempt to find the optimal way to integrate networks, that it assumes edge weights in different networks can be combined in a straightforward manner, that it does not discover novel terms, and that it cannot be evaluated using a training-testing procedure. Another recent study has attempted to extend existing GO by using biological networks^14^, but the method cannot infer GO automatically. Finally, there is a method that groups related terms based on genes that they annotate^15^, which can also discover term-term relationships as we do, but does not aim at inferring novel terms or constructing the ontology.

In the followings we describe the details of Unicorn and the empirical tests we have performed using data from *S. cerevisiae*.

## Methods

**Figure 1:**
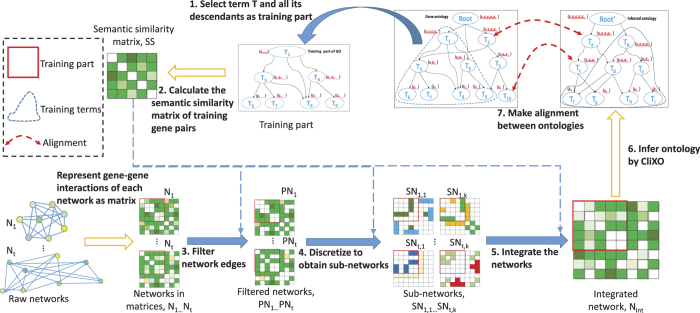
The overall pipeline of Unicorn for integrating multiple heterogeneous networks and inferring a GO sub-ontology. In the matrix representation of each network, a darker color indicates a larger value. Diagonal entries are ignored by CliXO and are always set to 0. The colors of the matrix entries after Step 4 are the new edge weights after discretization (explained in Fig. S4).

The overall pipeline of Unicorn for integrating multiple biological networks and inferring a GO sub-ontology is illustrated in Fig. 1. There are seven main steps, the details of which will be given in the corresponding sections below. Step 1: A sub-tree of a GO sub-ontology is selected as the training part. Step 2: For every pair of genes both annotated by a term in the training part (a “training gene pair”), their similarity in the sub-ontology is computed based on a simplified version^12^ of the Resnik semantic similarity measure^16^. Step 3: For each biological network, the edges are filtered based on the ontological similarity values of the training gene pairs, with a goal of removing edges irrelevant to the GO sub-ontology. Step 4: The weights of the retained edges are discretized in a concerted manner such that the different networks can be easily integrated. Step 5: The discretized networks are integrated to maximize the correlation between the discretized edge weights in the integrated network and the ontological similarity values of the training gene pairs. Integrating networks in this way is expected to make the resulting edge weights of the gene pairs not in the training set (the “left-out gene pairs”) useful for inferring their ontological relationships. Step 6: The CliXO method^12^ is run on the integrated network to infer a DAG based on all the genes. Step 7: The terms in the inferred DAG and the actual DAG of the GO sub-ontology are aligned^11^ to evaluate the similarity of the two DAGs based on the left-out gene pairs, and to discover novel terms in the inferred DAG.

The first 5 steps are novel to Unicorn, at the end of which a single integrated biological network is created and supplied as the standard input to CliXO. The semi-supervised nature of Unicorn allows it to make good use of the information in existing GO as compared to CliXO (Table S1).

### Selection of training part (Step 1)

There are two key considerations when choosing the training part from a GO sub-ontology, namely 1) the size of it should be big enough to capture sufficient information for guiding the network filtering, discretization and integration steps, and 2) the left-out part should not be too fragmented for otherwise it would be difficult to have terms in the inferred ontology that are not directly due to the training part, thereby making it hard to evaluate the effectiveness of Unicorn objectively. Consequently, for each GO sub-ontology, we select every sub-tree with a root between the 2nd and 5th levels as a training part, and use each of them to infer a DAG in turn.

### Filtering edges in biological networks (Steps 2 and 3)

Existing biological networks contain a lot of interactions discovered by high-throughput experiments, including some low-confidence interactions that could be false positives. There are also interactions irrelevant to the target GO sub-ontology. To prevent these interactions from misleading the ontology inference process, previous studies have filtered them using arbitrary edge weight thresholds or requiring each network to have the same final number of interactions^11^,^12^. In our semi-supervised pipeline, we instead use the training part to determine an appropriate threshold for each network individually.

We first compute an ontological similarity value for each training gene pair. As in a previous study^12^, we define the similarity between two genes *g*_*a*_ and *g*_*b*_ by a simplified Resnik measure based on the training part of the target sub-ontology:


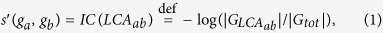


where *LCA*_*ab*_ is the lowest common ancestor term of genes *g*_*a*_ and *g*_*b*_ in the training part of the GO DAG, *IC*(*LCA*_*ab*_) is its information content, 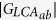 is the number of genes annotated by this lowest common ancestor term, and |*G*_*tot*_| is the total number of genes annotated by the terms in the training part (the “training genes”). A normalized score between 0 and 1 is then defined by dividing the simplified Resnik score by its maximum possible value: 
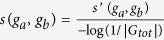
. Basically, if two genes are commonly annotated by a term that does not also annotate many other genes, they will receive a large similarity value based on this measure.

Gene pairs receiving a normalized score no less than a threshold *t*_*s*_ are considered semantically similar. Throughout the whole study, we set *t*_*s*_ to 0.3, which roughly corresponds to defining two genes as similar if they are commonly annotated by a term that annotates no more than 500 genes.

We then use these pairs of similar genes to filter the edges in each biological network (including both the training and left-out gene pairs), such that a large fraction of the retained edges are between semantically similar genes. Specifically, for each network, we retain only edges with an edge weight no smaller than a threshold *t*_*w*_, defined as the smallest value that leads to at least 50% of the retained training edges being semantically similar:


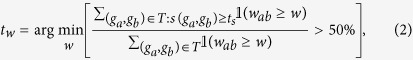


where *w*_*ab*_ is the weight of the edge between gene *g*_*a*_ and gene *g*_*b*_ in the network, *T* is the set of training gene pairs, and 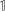 is the indicator function, i.e., 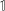
(*true*) = 1 and 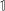
(*false*) = 0. Assuming that the general relationships between network edge weights and ontological similarity values are the same for the training and left-out gene pairs, this filtering can effectively retain only the more relevant network edges in the left-out part for inferring the DAG of the sub-ontology. The reason to search for the smallest *w* that satisfies the requirement in Eq (2) is to retain as many edges in the network relevant to the GO sub-ontology as possible. To identify this *t*_*w*_, we set *w* to the largest edge weight in the whole network at the beginning, and progressively reduce it to the next largest edge weight until the requirement in Eq (2) is satisfied. To handle the issue that the requirement in Eq (2) sometimes cannot be satisfied, or can only be satisfied with an extremely large value of *t*_*w*_, if the percentage of semantically similar training gene pairs does not increase for 5 consecutive reductions of *w*, the value of *w* before these 5 reductions would be used as *t*_*w*_.

### Unification of heterogeneous networks by discretizing edge weights (Step 4)

Before the filtered networks can be integrated, one issue that we need to first handle is the very different distributions of edge weight values in these different networks. We have tried various standard ways to process these values, such as linearly scaling all edge weights to the range of 0 to 1. However, the resulting distributions of the different networks were still very different, and direct integration of these networks would place more emphasis on the networks with more edge weights closer to 1. On the other hand, methods such as quantile normalization destroy the original distribution of edge weights in each network and led to serious information loss.

We found a good strategy to unify these heterogeneous networks is to discretize the edge weights in each network into comparable numbers of discrete levels, such that 1) the order of edges based on their original weights is respected, and 2) for the training gene pairs, the consistency between their discretized weights and ontological similarity values is maximized.

To achieve these two goals, we designed a novel discretization algorithm. Given a set of training gene pairs and a biological network, the algorithm searches for a discretization *M* of the edge weights (i.e., a mapping of the original edge weights to the discrete levels) such that the following objective function is minimized:


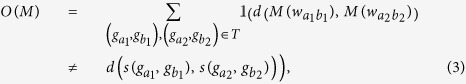


subject to the constraint that the order of the edges needs to be maintained, i.e., for any two training gene pairs 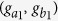 and 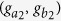, 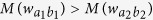 only if 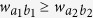 and 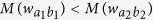 only if 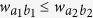. In the objective function, *d* is the direction function defined as *d*(*x, y*) = 1 if *x* > *y, d*(*x, y*) = 0 if *x* = *y* and *d*(*x, y*) = −1 if *x* < *y*. This objective function aims at minimizing the number of gene pairs that have different orders according to the discretized edge weight levels and according to their ontological similarity in the GO sub-ontology. Since in Step 6 of our pipeline CliXO is used to infer an ontology, and CliXO considers the order of edges in its clustering process rather than their absolute weights, our discretization procedure promotes gene pairs that are ontologically similar to be clustered earlier by CliXO.

We designed a searching algorithm to identify discretizations with a good objective score (Fig. S4). Initially, all training gene pairs are sorted based on their edge weights and random partition points are added to divide them into *k* (set to 200 by default) ordered levels, where gene pairs with the same edge weights must be put in the same level. The algorithm then repeatedly refines the levels by randomly either moving some top gene pairs of a level (i.e., gene pairs with the largest original edge weights) to the next higher level, or moving some bottom gene pairs to the next lower level. If the objective score is improved, the new discretization is kept; Otherwise, the discretization is kept with a probability that reduces over time (*min*{0.1/*iteration*_*number*, 0.001}), an idea similar to simulated annealing. The searching process stops after a maximum number of iterations (set to 10,000 by default), and the whole process is repeated multiple times using different random initial partitions. The discretization with the best objective score is then retrieved, and all edges in a level are given a new weight equal to the average of their original weights. Finally, some neighboring levels are combined to form 10–20 levels to avoid over-fitting in data integration step.

### Integration of multiple biological networks (Steps 5 and 6)

After discretizing the edge weights of each network individually, we integrate the networks by finding a linear combination of them that maximizes the Pearson correlation coefficient (*PCC*) with the semantic similarity values of the training gene pairs. Specifically, we find the coefficient vector **a** that maximizes *PCC*(**Ma**^*t*^, **s**) subject to the constraint that 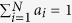, where *N* is the total number of merged discrete levels in the different networks, **M** is a |*T*| × *N* matrix of discretized edge values of the |*T*| training gene pairs, and **s** is a vector of the ontological similarity values of these training gene pairs. Each column in matrix **M** corresponds to one merged discrete level of one of the networks. Element (*i, j*) takes the value of the discretized edge weight of gene pair *i* if it belongs to the level represented by *j*, or 0 otherwise.

Since this optimization problem is a special case of canonical correlation analysis (CCA)^17^ for finding the most correlated linear combinations of two sets of variables, we use a standard routine for CCA in Matlab to determine the coefficients **a**.

After this step, all the biological networks are integrated into a single network with a new edge weight assigned to every (training and non-training) edge. This integrated network is then used as the input of CliXO to infer a DAG in which each node is formed by a cluster of genes and corresponds to a potential term in the target sub-ontology. CliXO is a hierarchical clustering method for grouping genes into potential terms. It starts by treating each gene as a node. Different nodes are merged to form a parent node of them if the genes contained in these nodes all have a similarity higher than a threshold with each other, where gene-gene similarity is defined based on the input biological network. If an edge is drawn between every two genes with a similarity higher than the threshold, each node is essentially a clique (i.e., a complete graph), which explains the name of the method (CliXO - Clique Extracted Ontology). The similarity threshold is set at a large value at the beginning, and is reduced progressively in rounds to allow more and more nodes to be merged together. CliXO also has additional steps to prune uninformative cliques and to allow for errors in the similarity values or imperfect cliques. These extra steps make the final output of CliXO not necessarily a tree, but a DAG in general.

### Data and Experiment Settings (Step 7)

#### Biological networks

We used four public yeast networks that had also been used for inferring GO in previous studies^11^,^12^, namely 1) correlation network of genetic interactions from DRYGIN (http://drygin.ccbr.utoronto.ca/DOWNLOAD/sgadata_costanzo2010_correlations.txt.gz)^18^, co-expression network from Stanford Microarray Database (SMD)(Provided by Michael Kramer)^19^, probabilistic functional gene network from YeastNet (v3)(http://www.inetbio.org/yeastnet/download.php?type=1)^20^, and network of physical interactions (of types “direct interaction” and “physical association”) from BioGRID (http://thebiogrid.org/downloads/archives/Release%20Archive/BIOGRID-3.3.122/BIOGRID-ORGANISM-3.3.122.mitab.zip)^21^. We considered only genes with at least one GO annotation. Some statistics of the four resulting networks are given in Table S2.

Since the edges in the BioGRID network were binary, we used a diffusion kernel^22^ to produce numeric edge weights between 0 and 1, which resulted in larger weights for genes more (directly or indirectly) connected to each other.

#### Gene ontology definition and annotation files

We downloaded the gene ontology and annotation files from the Gene Ontology Web site (http://geneontology.org/). We processed these files in the same way as in previous studies^11^,^12^ (Supplementary materials).

We downloaded two versions of GO ontology and annotation files. The first version (Ontology: 2-Dec-2014; Annotation: 29-Nov-2014) was the most updated version at the time we started the project and downloaded the files, which will be referred to as the 2014 version. The second version (Ontology: 31-Mar-2009; Annotation: 14-Mar-2009), which will be referred to as the 2009 version, represents an older version of GO that we used to test whether we could infer terms in the new version by combining the information in the old version and the biological networks. Some statistics of these two GO versions are given in Table S3. In addition to these two versions, in the part of our work that studied novel terms inferred by Unicorn, we also checked whether some of these terms were included in the latest version of GO at the time of paper writing (Ontology: 31-May-2016). This version will be referred to as the 2016 version.

For the 2014 version of GO, using the criteria we defined for selecting training parts described in Section ***Selection of training part***, we got 12, 12 and 9 training parts from BP, CC and MF, respectively.

#### Ontology alignment and performance evaluation

We used a slightly modified version (explained below) of the method described previously^11^ to align an ontology inferred by Unicorn with the actual GO sub-ontology. Briefly, a mapping of the terms in the two ontologies was produced to align highly similar terms based on the genes they annotate, with the constraints that 1) each term in the inferred ontology could be aligned to at most one term in the GO sub-ontology, and 2) the aligned term pairs could not crisscross. We used a false discovery rate of 5% as the cutoff to define a pair of terms to be aligned.

To objectively evaluate the performance of our inferred ontology using information not involved in the training process, we designed the following evaluation procedure (Fig. S1). Given a target GO sub-ontology *O*_*G*_ and a chosen sub-tree of it *O*_*T*_, we first inferred an ontology *O*_*G*′_ from all genes using Unicorn with *O*_*T*_ as the training part. Next, we considered only the genes annotated by terms in *O*_*T*_ to infer another ontology *O*_*T*′_ using *O*_*T*_ as the training part, and aligned it with *O*_*G*′_. For any term in *O*_*G*′_ aligned to a term in *O*_*T*′_, we considered it a term inferred due to information directly from the training part. Finally, we aligned *O*_*G*′_ and *O*_*G*_, and used only the aligned terms in *O*_*G*′_ not considered to be due to the training part to evaluate the performance of the inferred ontology. Specifically, if *A*(*O*_*x*_, *O*_*y*_) is the set of aligned term pairs from ontologies *O*_*x*_ and *O*_*y*_, we defined *Hit* as the number of terms in *O*_*G*′_ aligned to *O*_*G*_ but not due to the training part, i.e., *Hit* = |{(*t*_*G*′_, *t*_*G*_) ∈ *A*(*O*_*G*′_, *O*_*G*_):*t*_*G*_ ∉ *O*_*T*_ ∧ ¬ ∃ *t*_*T*′_*s*.*t*.(*t*_*G*′_, *t*_*T*′_) ∈ *A*(*O*_*G*′_, *O*_*T*′_)}|. Three performance metrics were then defined accordingly:


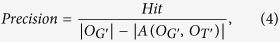



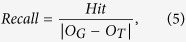



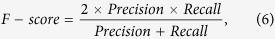


where |*O*_*x*_| is the number of terms in an ontology *x* and |*O*_*G*_ − *O*_*T*_| is the set of terms in the GO sub-ontology not in the training part.

In the original alignment algorithm^11^, the similarity between two terms from the two ontologies is based on both the genes they annotate (their “intrinsic similarity”) and their parent and child terms (their “hierarchical similarity”). In our case, when we aligned *O*_*G*′_ and *O*_*G*_, some of the parent/child terms were those considered to be due to the training part. In order to remove any effects of the training part in our performance evaluation, we modified the alignment algorithm to consider only the intrinsic similarity between two terms in all our experiments.

## Results

### Edge filtering increased fraction of informative edges

In the filtering step of Unicorn (Step 3), some edges are removed such that among the training gene pairs with a retained edge, a larger fraction of them are informative (i.e., having an ontological similarity larger than threshold *t*_*s*_) after the filtering. We checked whether the filtering also increased the fraction of informative edges among the left-out genes as judged by their actual ontological similarity according to the GO sub-ontology (which was not disclosed to Unicorn). As shown in Table 1, indeed for all three GO sub-ontologies and all four biological networks, the filtering increased the fraction of informative edges among left-out gene pairs, thus verifying the effectiveness of the filtering step.

### Unicorn improved accuracy of ontology inference

We then checked the accuracy of ontology inference of Unicorn based on the left-out parts. CliXO contains two key parameters, namely *α* (for reducing noise by adding a margin to the similarity threshold when forming cliques) and *β* (for inferring missing edges by allowing near-complete graphs as new terms). We set *β* to 0.5 as previously suggested^12^, and varied the value of *α* such that each set of results contained points from one extreme (high precision, low recall) to the other extreme (high recall, low precision).

**Figure 2:**
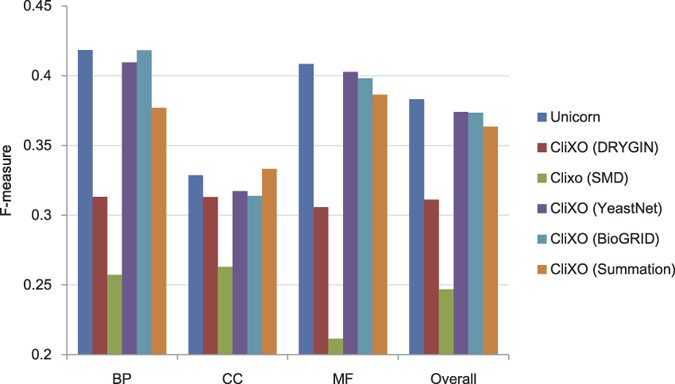
The average F-measures of CliXO with either Unicorn-produced integrated network, a single biological network, or a simple summation of the input networks. Each reported F-measure is the average among the results from all the training parts of one GO sub-ontology (in the case of “BP”, “CC” and “MF”) or across all three sub-ontologies (in the case of “Overall”), over all parameter values of CliXO.

Figure 2 shows the overall F-measure of Unicorn as compared to running CliXO on individual networks and a simple benchmark method, averaging over the parameter values. In this benchmark method, the weight of an edge in the integrated network is simply the summation of its weight in the original networks, which assumes equal importance of the input networks. It is seen that when inferring BP, information from BioGRID was most useful followed by YeastNet. On the other hand, when inferring CC, YeastNet was most useful followed by DRYGIN and BioGRID. Finally, when inferring MF, YeastNet was most useful followed by BioGRID. These results indicate that the different networks should be integrated differently when inferring the three sub-ontologies. Indeed, a simple summation of the four networks led to improved F-measure only for CC but not in the cases of BP, MF and the overall average. On the other hand, by having a semi-supervised framework that processes and integrates the networks specific to the target GO sub-ontology, Unicorn was able to achieve better average F-measure values both when inferring each sub-ontology and averaging over all three sub-ontologies overall, as compared to using individual networks as input.

**Figure 3:**
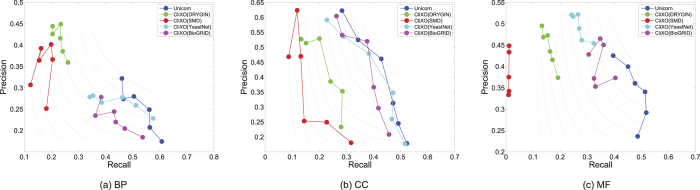
Ontology inference left-out accuracy of Unicorn and single biological networks. The training parts were the sub-trees rooted at GO:0051179 (localization), GO:0016020 (membrane) and GO:1901363 (heterocyclic compound binding) for BP, CC and MF, respectively.

Figure 3 shows some examples of the comparison results in the form of precision-recall graphs. In each graph, each approach is represented by a curve joining different points that correspond to the results when running CliXO with different *α* values. The dotted curves in the background are contour lines that connect points with the same F-measure score. From the graphs, the left-out parts of the ontologies inferred by Unicorn have higher F-scores in general, as seen by their positions closer to the upper-right corner.

**Figure 4:**
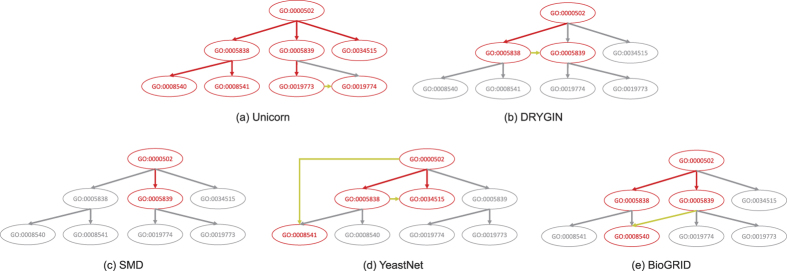
Ability to infer the sub-tree rooted at GO:0000502 (proteasome complex) by single biological networks and Unicorn with the sub-tree rooted at GO:0043226 (organelle) as the training part. The colors represent successfully inferred (red), missed (gray) and novel (yellow) terms and term-term relationships as compared to the 2014 version of GO.

Figure 4 gives an example illustrating the importance of integrating the biological networks. It shows the ability of different networks in inferring the sub-tree of the CC sub-ontology rooted at the CC term GO:0000502 (proteasome complex) when the sub-tree rooted at the term GO:0043226 (organelle) was used as the training part. As seen in the figure, while each individual network was sufficient to infer part of the sub-tree, only when the networks were integrated was it possible to infer all the terms.

### Re-discovering terms in new version of GO by combining information in an old version of GO with biological networks

While the above results have confirmed the accuracy of Unicorn using left-out parts of GO not involved in the training process, the ultimate use of Unicorn is to infer novel terms not already contained in GO. The first way we attempted to test this possibility was to combine the information in the biological networks and an old (2009) version of GO, to see if Unicorn could infer terms that were only in a new (2014) version of GO.

**Figure 5:**
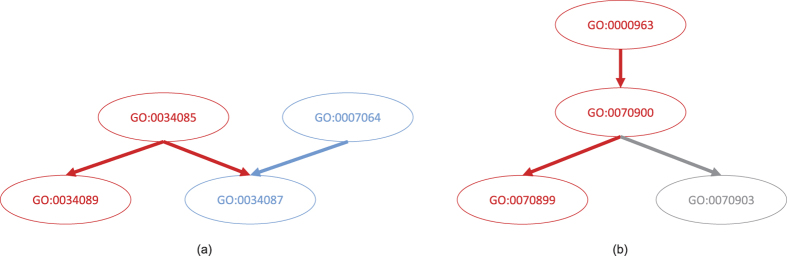
Some terms inferred by Unicorn by combining the information in the biological networks and the 2009 version of GO. The colors represent terms and term-term relationships only present in the 2014 version of GO but not the 2009 version (red), present in both the 2009 and 2014 versions of GO (blue), and absent in both the 2009 and 2014 versions of GO (gray), but present in the 2016 version. These two terms were all inferred from BP.

By running Unicorn with the 2009 version of GO as input, the inferred DAG contained nodes that could not be aligned to any term in this version of GO. Based on the CliXO procedure, each of these nodes contained a set of genes and was connected to other nodes in the inferred DAG. Each such node can therefore be considered a potential novel term that annotates these genes and are related to other existing terms in the 2009 version of GO based on their connections in the inferred DAG. We then aligned all the terms in the inferred DAG with the 2014 version of GO, and found some of the nodes not aligned to the 2009 version of GO actually aligned to some nodes in the 2014 version. Specifically, we identified 3–19, 6–10 and 1–6 cases in BP, CC and MF, respectively for different values of *α* when running CliXO. Figure 5 and Fig. S2 show some of the examples.

In these examples, we see that Unicorn is able to infer both general (upper-level) and specific (lower-level) terms present only in the 2014 version of GO. It is possible that some of the Unicorn-inferred terms that cannot be aligned to either the 2009 or 2014 version of GO (the ones in gray) are biologically meaningful novel terms. In fact, for some of them (the nodes in gray with GO term IDs) we actually find nodes in the 2016 version of GO connecting to the corresponding parent and child terms as in our inferred DAG. For the remaining novel terms, we further explore their potential meanings in the next section.

### Discovery of biologically meaningful novel terms

**Figure 6:**
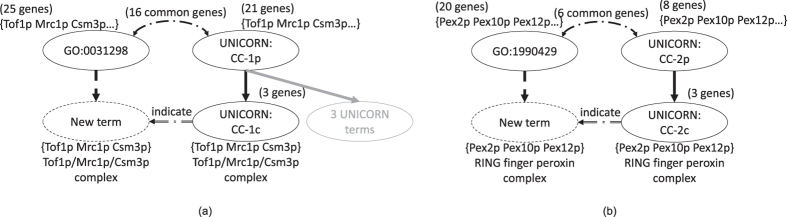
Some biologically meaningful novel terms inferred by Unicorn. In each panel, the terms on the right were inferred by Unicorn.

Unicorn inferred a large number of novel terms not contained in either the 2009, 2014 or 2016 version of GO. To investigate their potential meanings, we extracted the list of genes annotated by them and looked for descriptions of these gene groups in the literature. Some examples with supports from the CYC 2008 protein complex database^23^ are shown in Fig. 6.

In the first example (Fig. 6a), Unicorn identified a sub-complex of the replication fork protection complex (GO:0031298) involving three proteins Csm3p, Mrc1p and Tof1p. These three proteins form the replication fork-pausing complex (FPC)^24^,^25^, which is associated with replication sites and prevents genomic instability through mediating checkpoint signaling in stationary-phase cells^26^.

In the second example (Fig. 6b), Unicorn identified a sub-complex of the peroxisomal importomer complex (GO:1990429) involving three proteins Pex2p, Pex10p and Pex12p. These three proteins form the RING finger peroxin complex^27^,^28^,^29^, which was considered to function in peroxisomal matrix protein import by recycling receptors^27^.

Six additional novel terms are shown in Fig. S3 and their literature supports are given in the supplement. We also provide on our supplementary Web site a list of unverified novel terms with either a parent or child term aligned to a GO term with score >0.8.

## Discussion

In this paper, we proposed a semi-supervised framework to integrate multiple biological networks for better automatic inference of Gene Ontology. The results based on the left-out parts of GO not involved in training confirmed the accuracy of the inferred ontologies. The better performance of Unicorn as compared to CliXO in some of the experimental results were due to the semi-supervised nature of Unicorn, which allowed it to integrate both the information in the biological networks and in the training part of GO. These training data helped Unicorn to 1) determine the most relevant network edges to retain, 2) discretize network edges such that multiple heterogeneous networks can be easily integrated, and 3) determine the best way to integrate these networks by maximizing the correlation between the edge weights in the resulting integrated network and the ontological similarity of the training part. All these novel components contributed to the construction of an integrated network more suitable for CliXO to infer GO from.

We were also able to rediscover terms in a new (2014) version of GO based on information in an old (2009) version, and discover novel terms that were shown to be biologically meaningful. Unicorn can thus be used to propose new terms for further manual validation and curation.

We selected four biological networks in our study based on the successful use of them in inferring GO in some previous work^11^,^12^. We showed that these four networks contributed unequally, and for each GO sub-ontology the way to use them should be customized, which highlights the advantage of a supervised or semi-supervised approach as compared to previous unsupervised approaches.

It is useful to explore the integration of more types of biological network such as those based on the evolutionary relationships of the genes, and the possibility to apply Unicorn to other species and other types of biological ontology.

One of the main uses of GO is functional enrichment analyses. The DAGs constructed by Unicorn provide a putative set of terms potentially useful for explaining the functional relationships between some genes. An advantage of using these Unicorn-constructed terms is that the molecular basis of them can be easily traced back from the similarity of the genes in the integrated network, with the importance of each network indicated by its respective coefficient in the integration formula.

## Additional Information

**How to cite this article**: Li, L. and Yip, K. Y. Integrating Information in Biological Ontologies and Molecular Networks to Infer Novel Terms. *Sci. Rep.* **6**, 39237; doi: 10.1038/srep39237 (2016).

**Publisher’s note:** Springer Nature remains neutral with regard to jurisdictional claims in published maps and institutional affiliations.

## Supplementary Material

Supplementary Materials

## Figures and Tables

**Table 1:** Average fraction of informative edges in the biological networks among the left-out genes before and after edge filtering.

		DRYGIN	SMD	YeastNet	BioGRID
BP	Before filtering	11.75%	19.20%	40.44%	40.39%
	After filtering	47.79%	70.43%	67.84%	53.41%
CC	Before filtering	3.47%	7.17%	21.43%	26.75%
	After filtering	41.38%	73.46%	59.62%	58.00%
MF	Before filtering	3.73%	5.46%	16.05%	13.42%
	After filtering	15.66%	74.70%	57.23%	32.90%

These values were obtained by averaging over all the training parts of each sub-ontology.
