# Supplementary material for: Integrating Information in Biological Ontologies and Molecular Networks to Infer Novel Terms
Source: Sci Rep. 2016 Dec 15;6:39237. doi: 10.1038/srep39237 (PMC5157009; doi:10.1038/srep39237)
Supplement: Supplementary Materials [file srep39237-s1.pdf]

Supplementary materials for Integrating Information in Biological Ontologies and Molecular Networks to Infer Novel Terms

Le Li[1] **and Kevin Y. Yip**[1,2,3,4*]

[1]Department of Computer Science and Engineering,
[2]Hong Kong Bioinformatics Centre,
[3]CUHK-BGI Innovation Institute of Trans-omics, and
[4]Hong Kong Institute of Diabetes and Obesity, The Chinese University of Hong Kong, Shatin, New Territories, Hong Kong
[*]kevinyip@cse.cuhk.edu.hk

Supplementary methods

Following Dutkowski et al. (2013)[1], the Gene Ontology term definition and gene annotation files were processed as follows:

- Terms labeled as 'is_obsolete' were removed.
- For any pair of terms (x, y), if their relationship was specified as either 'x is_a y', 'x part_of y', 'y has_part x', 'x regulates y', 'x positively_regulates y' or 'x negatively_regulates y', an edge was drawn from the node that represents x to the node that represents y.
- Annotations labeled as 'NOT' were removed.
- Only genes contained in at least one of the biological networks were considered.
- If a gene was annotated by a term, it was also considered as annotated by all ancestors of the term[2] according to the True Path Rule[3].
- Terms annotating zero or only one gene were discarded since there would be no way to recover such terms by CliXO[4].
- If a term x and a child term of it y annotated exactly the same set of genes, x would be removed and the y would inherit all its informative relations. All other child terms of x would become child terms of y.

Supplementary results

1. Vac14p and Fig4p were shown to form a complex[5,6] that regulates phosphatidylinositol 3.5-bisphosphate synthesis and turnover, which is part of the function of the PAS complex (GO:0070772).
2. Gyp5p and Gyl1p were considered to form a complex[7,8], which co-purifies with post-Golgi vesicles[7] and is thus related to GO:0005798 (Golgi-associated vesicle).
3. Crh1p and Bug1p form a complex at the cis-Golgi network (GO:0005801)[9,10], and it was suggested to contribute to a redundant network of interactions that mediate consumption of COPII vesicles and formation of the cis-Golgi[9].
4. Bi4p and Nam2p form a complex required for splicing bI4 of the yeast COB gene[11], which suggests that the Bi4p/Nam2p complex should be associated with GO:0000372 (Group I intron splicing).
5. Coq3 and Coq4 form the Q-biosynthetic Coq polypeptide complex[12], which was verified to exist in yeast mitochondria for the biosynthesis of coenzyme Q (ubiquinone)[13]. This suggests the inferred term should be related to GO:0006744 (Ubiquinone biosynthetic process).
6. Sur1p and Csg2p form a sub-complex of the Sur1p/Csg2p/Csh1p peroxisomal importomer complex (GO:1990429) with an unknown function, and was required for growth of yeast under high calcium concentrations[14].

Supplementary tables

Method	CliXO	Unicorn
Type	Unsupervised	Semi-supervised
Filtering of network edges	Fixed thresholds	Thresholds learned from training part of GO
Unification of heterogeneous networks	Nil	By means of discretization
Integration of networks	Fixed scheme	Weights learned from training part of GO

Table S1. Major differences between the CliXO and Unicorn methods

Network	DRYGIN	SMD	YeastNet	BioGRID
Genes (before filtering)	3,919	4,627	5,805	5,557
Edges (before filtering)	6,442,244	2,906,334	361,984	69,680
Edge weight type	continuous	continuous	continuous	binary
Edge weight range	(0,1)	(0,1)	(0.934,5.740)	{0,1}
Genes* (after filtering)	2,142	2,102	4,924	4,589
Edges* (after filtering)	6,349	18,183	54,332	28,916

Table S2. Statistics of the four yeast biological networks (*Average number for the different target sub-ontologies and training parts considered)

Version		2014	2009
BP	Terms	2,850	2,146
	Genes annotated	5,910	5,898
	Term-term relationships	7,078	4,297
CC	Terms	734	617
	Genes annotated	5,912	5,898
	Term-term relationships	1,721	1,363
MF	Terms	1,252	1,619
	Genes annotated	5,909	5,896
	Term-term relationships	1,740	1,998

Table S3. Statistics of the 2009 and 2014 versions of GO used in the experiments

Supplementary figures

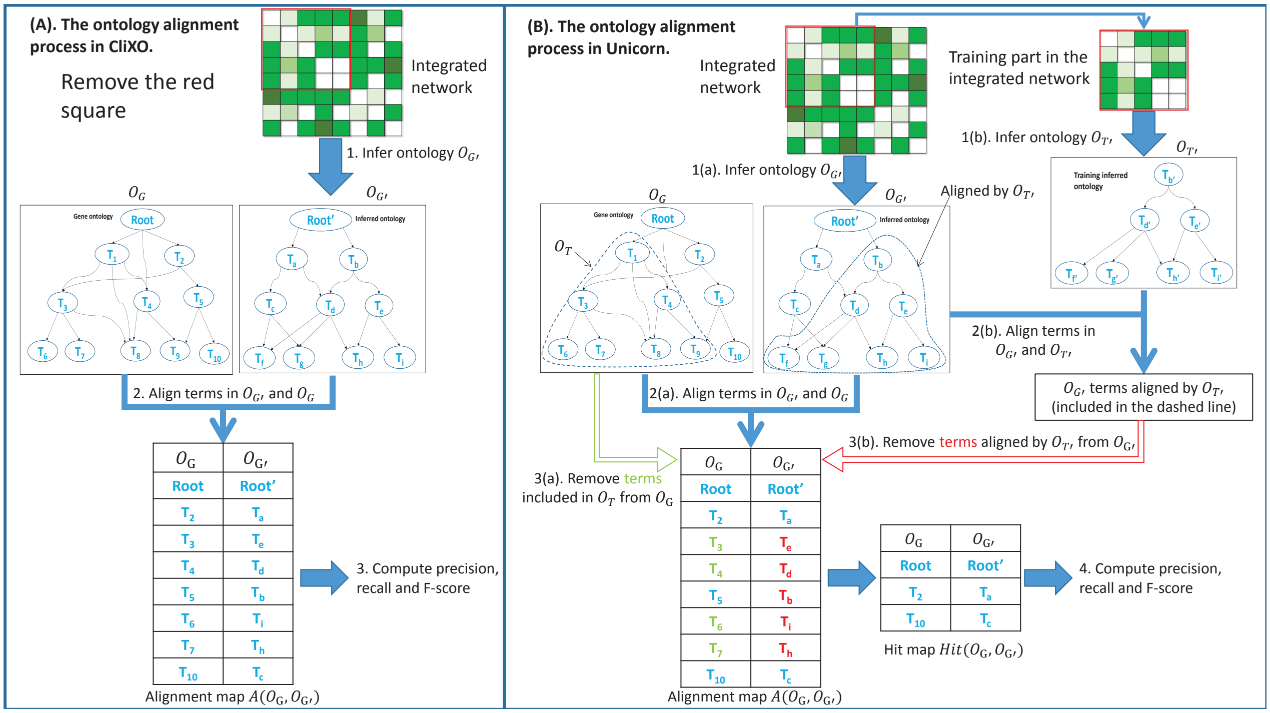

Figure S1. The training-testing procedure for evaluating the effectiveness of Unicorn. Panel A shows the procedure for evaluating an ontology inferred by CliXO without going through the Unicorn process. The input network (either a single biological network or an integrated network) is used to infer an ontology $O_{G'}$, the terms of which are aligned with the terms in the actual GO sub-ontology O_G. Based on the number of aligned terms and the total number of terms in $O_{G'}$ and O_G, precision, recall and F-measure can be computed. In contrast, Panel B shows the modified procedure for ontologies inferred with Unicorn. In this case, the training part is used to learn the parameters for filtering, unifying and integrating biological networks. The resulting integrated network, involving both the training and left-out parts, is used to infer an ontology $O_{G'}$. The terms of it are aligned to the terms in the actual GO sub-ontology O_G. However, instead of using the alignment results to evaluate the effectiveness of Unicorn directly, terms in $O_{G'}$ that are likely due to the training part are first removed (see main text for the details), before the resulting list of aligned term pairs is used to compute the performance metrics.

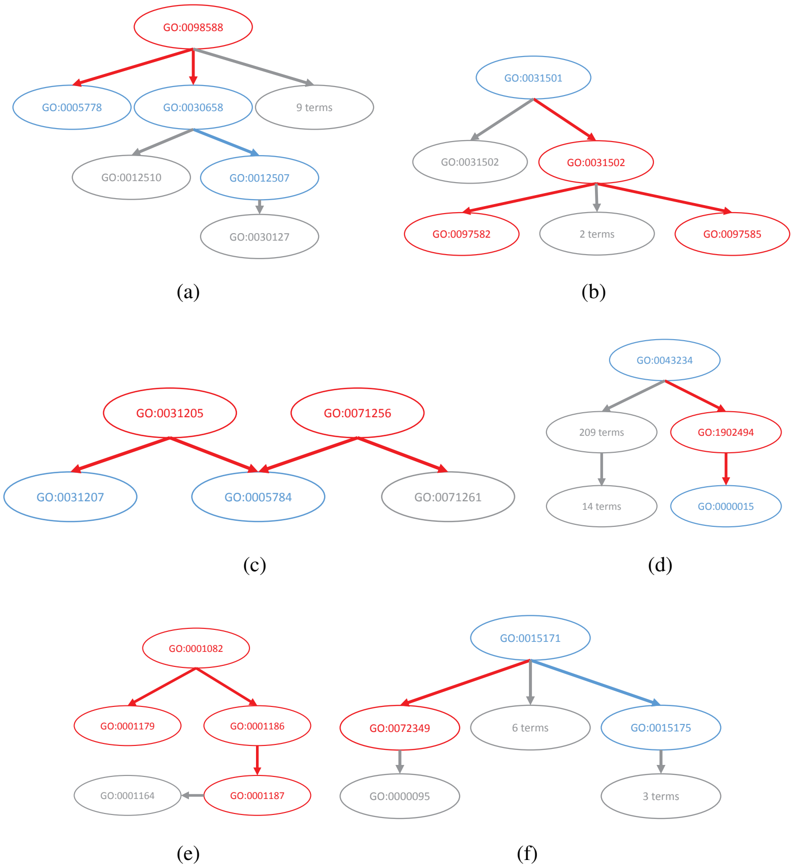

Figure S2. Some terms inferred by Unicorn by combining the information in the biological networks and the 2009 version of GO. The colors represent terms and term-term relationships only present in the 2014 version of GO but not the 2009 version (red), present in both the 2009 and 2014 versions of GO (blue), and absent in both the 2009 and 2014 versions of GO (gray). Some of the terms absent in both the 2009 and 2014 versions are present in the 2016 version, and the corresponding Go term IDs are shown. These terms were inferred from CC (a-d) and MF (e-f), respectively.

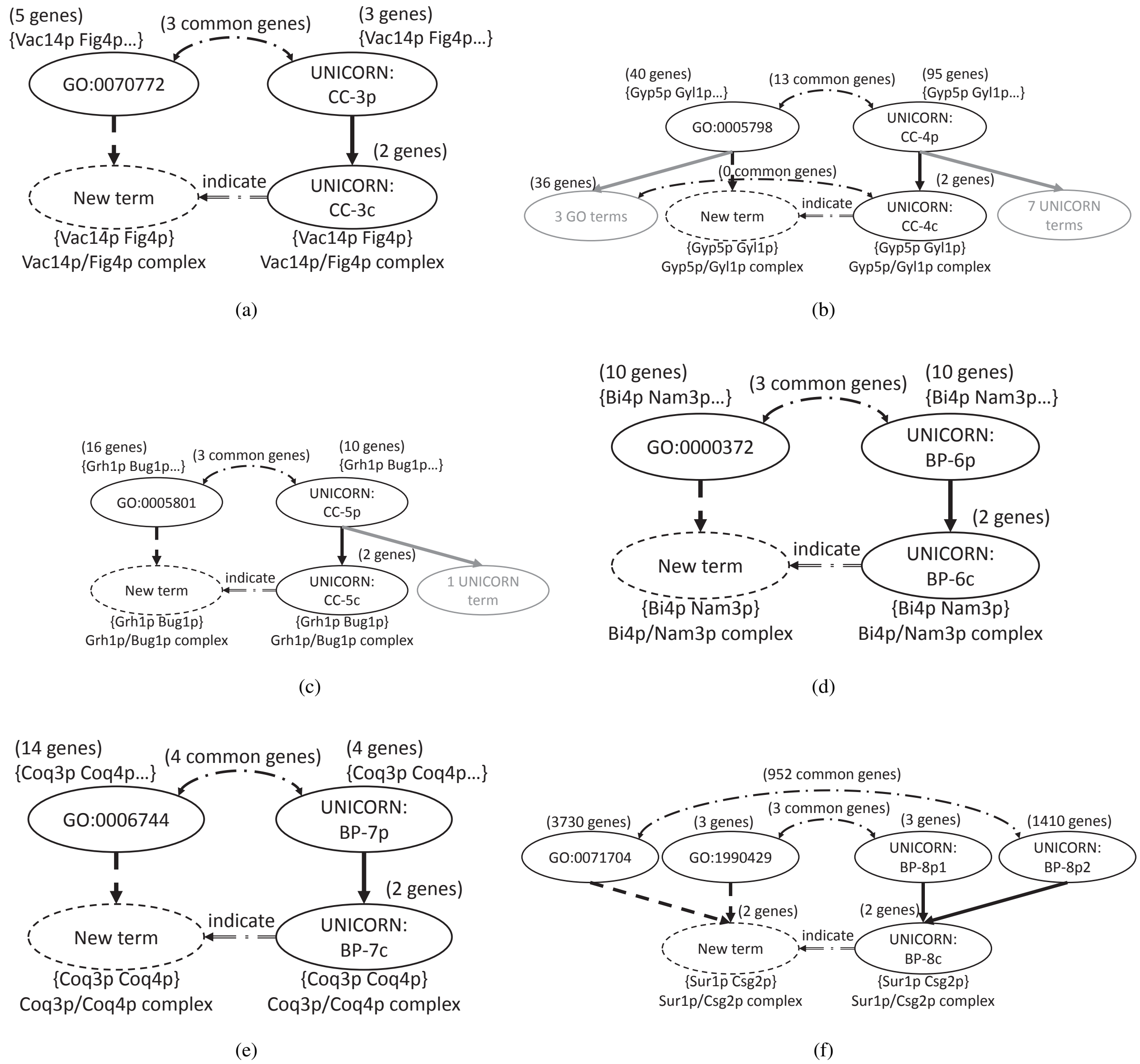

Figure S3. Additional biologically meaningful novel terms inferred by Unicorn. In each panel, the terms on the right were inferred by Unicorn.

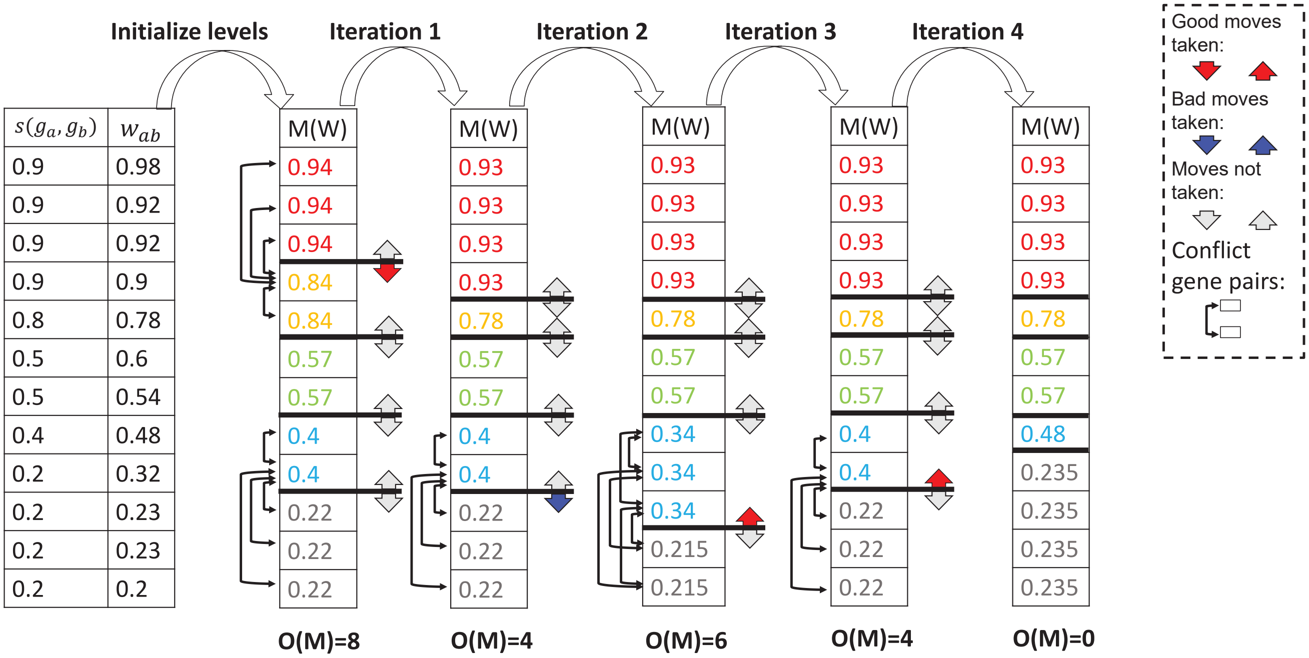

$s(g_a, g_b)$	w_{ab}
0.9	0.98
0.9	0.92
0.9	0.92
0.9	0.9
0.8	0.78
0.5	0.6
0.5	0.54
0.4	0.48
0.2	0.32
0.2	0.23
0.2	0.23
0.2	0.2

M(W)	M(W)	M(W)	M(W)	M(W)
0.94	0.93	0.93	0.93	0.93
0.94	0.93	0.93	0.93	0.93
0.94	0.93	0.93	0.93	0.93
0.84	0.93	0.93	0.93	0.93
0.84	0.78	0.78	0.78	0.78
0.57	0.57	0.57	0.57	0.57
0.57	0.57	0.57	0.57	0.57
0.4	0.4	0.34	0.4	0.48
0.4	0.4	0.34	0.4	0.235
0.22	0.22	0.34	0.22	0.235
0.22	0.22	0.215	0.22	0.235
0.22	0.22	0.215	0.22	0.235

Figure S4. An example illustrating the discretization process. Each row in the tables corresponds to one training gene pair. The thick horizontal bars represent the level partitions. At the beginning, the four randomly added partitions divide the training gene pairs into five levels. Each gene pair in a level receives a new edge weight equal to the average of the original edge weights of all the pairs in that level. This initial partitioning has an objective score of $O(M) = 8$, and the 8 corresponding pairs of conflicting gene pairs are indicated in the figure. Then, each partition can either move up or down (assuming moving step of 1 gene pair in this example, but the step size is arbitrary in the actual algorithm). If a move leads to an improved (i.e., reduced) objective score (indicated by a red arrow), the move will be taken. Otherwise, it will be taken with a certain probability (indicated by a blue arrow), and not taken otherwise (indicated by a gray arrow). In this example, after 4 iterations, the object score improves from 8 to 0. In the actual algorithm, some of the neighboring levels will be further merged, which is not shown in this example.

References

1. Dutkowski, J. *et al.* A gene ontology inferred from molecular betworks. *Nature Biotechnology* **31**, 38–45 (2013).
2. Huntley, R. P., Sawford, T., Martin, M. J. & O'Donovan, C. Understanding how and why the gene ontology and its annotations evolve: the GO within UniProt. *Gigascience* **3**, 4 (2014).
3. Gene Ontology Consortium. Creating the gene ontology resource: Design and implementation. *Genome Research* **11**, 1425–1433 (2001).
4. Kramer, M., Dutkowski, J., Yu, M., Bafna, V. & Ideker, T. Inferring gene ontologies from pairwise similarity data. *Bioinformatics* **30**, i34–i42 (2014).
5. Dove, S. K. *et al.* Vac14 controls ptdins (3,5)p(2) synthesis and fab1-dependent protein trafficking to the multivesicular body. *Current Biology* **12**, 885–893 (2002).
6. Rudge, S. A., Anderson, D. M. & Emr, S. D. Vacuole size control: Regulation of ptdins (3,5)p(2) levels by the vacuole-associated vac14-fig4 complex, a ptdins(3,5)p(2)-specific phosphatase. *Molecular Biology of the Cell* **15**, 24–36 (2004).
7. Chesneau, L. *et al.* Gyp5p and gyl1p are involved in the control of polarized exocytosis in budding yeast. *Journal of Cell Science* **117**, 4757–4767 (2004).
8. Chesneau, L. *et al.* Interdependence of the ypt/rabgap gyp5p and gyl1p for recruitment to the sites of polarized growth. *Traffic* **9**, 608–622 (2008).
9. Behnia, R., Barr, F. A., Flanagan, J. J., Barlowe, C. & Munro, S. The yeast orthologue of GRASP65 forms a complex with a coiled-coil protein that contributes to ER to golgi traffic. *The Journal of Cell Biology* **176**, 255–261 (2007).
10. Čopič, A. *et al.* Genomewide analysis reveals novel pathways affecting endoplasmic reticulum homeostasis, protein modification and quality control. *Genetics* **182**, 757–769 (2009).
11. Rho, S. & Martinis, S. A. The bi4 group i intron binds directly to both its protein splicing partners, a tRNA synthetase and maturase, to facilitate RNA splicing activity. *RNA* **6**, 1882–1894 (2000).
12. Pu, S., Wong, J., Turner, B., Cho, E. & Wodak, S. J. Up-to-date catalogues of yeast protein complexes. *Nucleic Acids Research* **37**, 825–831 (2009).
13. Marbois, B. *et al.* Coq3 and coq4 define a polypeptide complex in yeast mitochondria for the biosynthesis of coenzyme q. *Journal of Biological Chemistry* **280**, 20231–20238 (2005).
14. Jo, W. J. *et al.* Identification of genes involved in the toxic response of saccharomyces cerevisiae against iron and copper overload by parallel analysis of deletion mutants. *Toxicological sciences* **101**, 140–151 (2007).
